# Molecular Investigation of the Transmission Pattern of *Brucella suis* 3 From Inner Mongolia, China

**DOI:** 10.3389/fvets.2018.00271

**Published:** 2018-10-29

**Authors:** Zhi-guo Liu, Li-jun Wang, Dong-ri Piao, Miao Wang, Ri-hong Liu, Hong-yan Zhao, Bu-yun Cui, Hai Jiang

**Affiliations:** ^1^State Key Laboratory for Infectious Disease Prevention and Control, National Institute for Communicable Disease Control and Prevention, Chinese Center for Disease Control and Prevention, Beijing, China; ^2^Inner Mongolia Autonomous Region Center for Comprehensive Disease Control and Prevention, Huhhot, China; ^3^Hulun Buir People's Hospital of the Inner Mongolia Autonomous Region, Hohhot, China; ^4^Brucellosis Prevention and Treatment Engineering Technology Research Center of Inner Mongolia Autonomous Regeion, Tongliao, China

**Keywords:** transmission pattern, *Brucella suis* 3, MLVA, molecular investigation, inner mongolia

## Abstract

Brucellosis is an endemic disease in China affecting both humans and livestock. The aim of the present study was to analyze two *Brucella* strains isolated from sheep spleens from Ulanqab in Inner Mongolia, China using classical and molecular typing techniques. The two strains were identified as *Brucella suis* biovar 3 and were closely related to isolates previously obtained from two different hosts (human and swine) in Guangxi Province. Our results suggest that *B. suis* can be directly or indirectly transferred from swine to sheep, which act as reservoirs for *B. suis* infection and later transmitted to humans. Multiple locus variable-number tandem repeat analysis (MLVA) is a useful tool for tracing the geographical origin of brucellosis infections and elucidating its transmission patterns.

## Background

Brucellosis is a zoonotic bacterial disease caused by *Brucella* spp., a gram-negative bacteria that infects a wide range of mammals, including domestic and wild animals and humans, resulting in severe economic loss (1, 2). *Brucella melitensis, Brucella abortus*, and *Brucella suis* have been reported to infect both animals and humans (3). Infected domestic and wild animals serve as the natural reservoir and source of infection, which occurs through direct contact or consumption of undercooked meat and other contaminated animal products (4).

Prevention of human brucellosis largely depends on disease control in animals (5). Upon isolation, it is essential to identify the Brucella species and biovar, which in turn are utilized in epidemiological follow-ups and control of disease. Identification of Brucella strains in livestock is also essential to importation (6). Classical biotyping methods remain the definitive diagnostic approach and gold standard for confirming and identifying *Brucella* spp. at the species and biovar levels (7), although it only provides limited epidemiological information. Due to technical difficulties, previous studies have mainly concentrated on Brucella infection surveillance in Inner Mongolia of China, and investigations on the biological significance of shifts in *B. suis* hosts are limited. The multiple locus variable-number tandem repeat analysis-16 (MLVA-16) scheme has been extensively utilized in resolving closely related isolates (8, 9). This method has been proven to be effective in generating epidemiological information on Brucella strains, particularly in identifying its geographical source and elucidating transmission patterns (10, 11). In the present study, the MLVA-16 scheme was used to characterize two *B. suis* isolates from the spleens of sheep from Inner Mongolia that were collected in 2017.

## Materials and methods

### Bacterial culture and isolation

Bacteriological analysis was performed at a biosafety level-3 facility at the Chinese Centre for Disease Prevention and Control, National Institute for Communication Disease Prevention and Control. Forty-one sheep spleens were subjected to bacterial culture. Brucella isolates were primarily identified using standard procedures as described elsewhere (12, 7). Two Brucella strains (ws151 and ws152) were isolated from the spleens of sheep from a slaughterhouse in 2016. Brucella suis 1330 was used as a biotyping control.

### Biotyping

All isolates were identified as Brucella species on the basis of classical identification procedures (13), including CO2 requirement and H2S production, inhibition of growth by basic fuchsin (20 μg/mL) and thionin (20 μg/mL), agglutination with monospecific antiserum for A and M antigens, and phage identification. Brucella monospecific antisera A and M were obtained from the Chinese Centre for Disease Prevention and Control, National Institute for Communication Disease Prevention and Control.

### DNA preparation and MLVA genotyping

A loopful of cultured bacterial cells was dissolved in double distilled water, and heat treated at 99°C for 20 min. Total genomic DNA was extracted using a bacterial DNA purification kit (Wizard Genomic DNA Purification Kit, Promega, USA), according to the manufacturer instructions and stored at −20°C until analysis. The two isolates were identified on the basis of the Suis-ladder PCR (14). Genotyping was performed using a combination of both minisatellite and microsatellite tandem repeats as previously described (11, 15, 16).

### Analysis of MLVA data

Sixteen VNTRs were divided into three panels (panels 1, 2A, and 2B) such as MLVA8 = panel 1, MLVA11 = panels 1 + 2A, MLVA16 = panels 1 + 2A + 2B. The total number of repeat units at each locus was determined based on amplicon size as previously described (15). The copy number at each locus and each panel was managed as a character dataset using BioNumerics version 6.6 (Applied Maths, Belgium). Cluster analysis was based on categorical coefficients and the unweighted-pair group method with arithmetic mean algorithm (UPGMA). MLVA-16 was used to compare the epidemiological relationships between strains of *B. suis* biovar 3 obtained from Ulanqab and 59 *B. suis* isolates from different geographical regions in China (Table S1). The MLVA-16 primers used in this study are presented in Table S2. MLVA profiles of two strains in this study have been submitted to the MLVA bank 2016. (http://microbesgenotyping.i2bc.paris-saclay.fr/databases).

## Results

**Figure 1:**
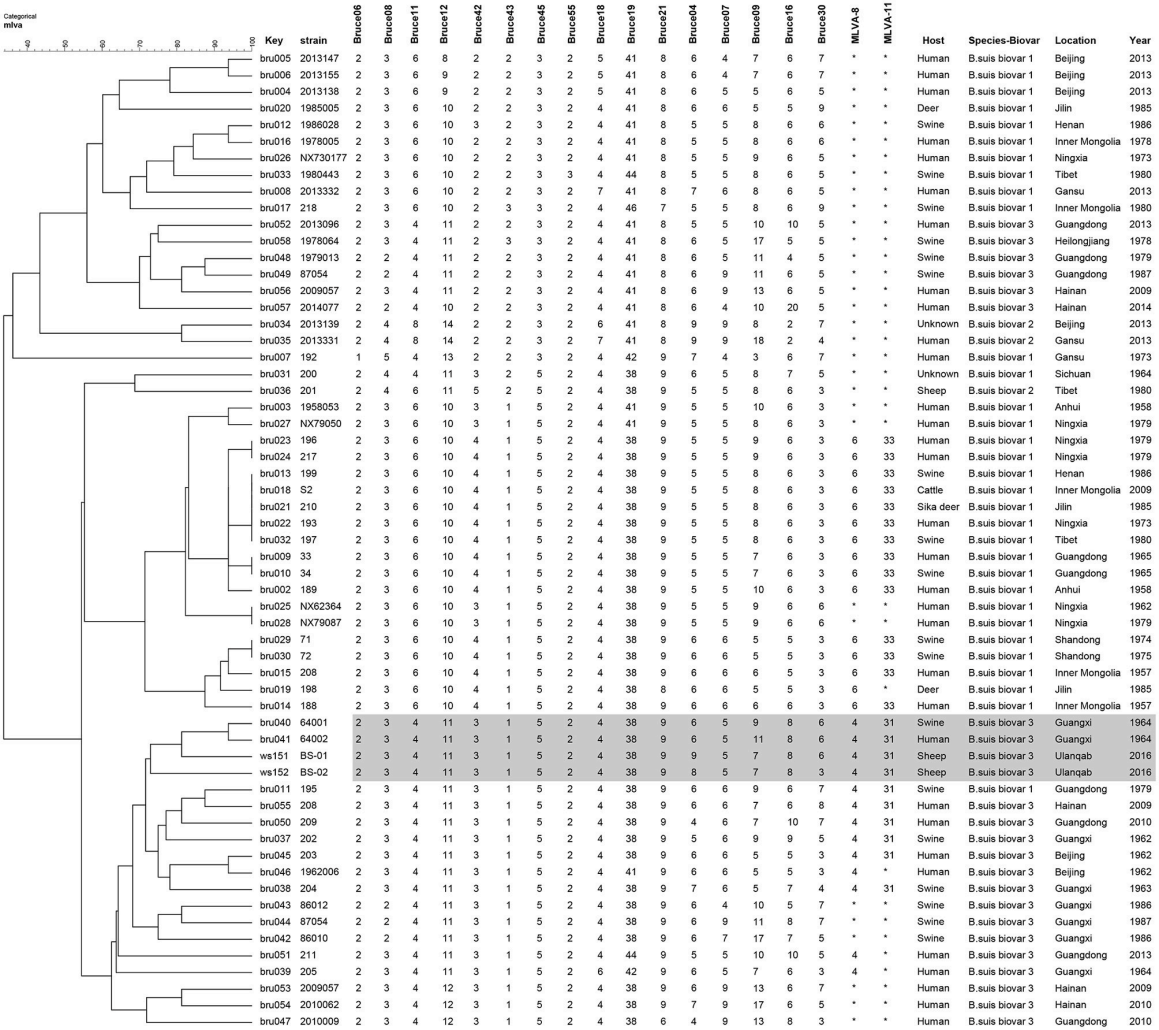
Dendrogram based on the MLVA-16 genotyping assay showing the relationship between the two *B. suis* isolates in this study and 67 other *B. suis* biovars from China. The rows highlighted in gray indicate that these have similar MLVA-16 genotypes.

In the present study, two Brucella strains were isolated from Ulanqab of Inner Mongolia in 2016. Colony morphology, staining, growth features, and slide agglutination with monospecific anti-Brucella sera were used to characterize the two isolates (Table 1). Using standard bacteriological procedures, the isolates were classified at the species and biovar levels. The profiles of the two isolated strains matched that of *B. suis*, and classical biotyping indicated that the *B. suis* isolate was of biovar 3. Two *B. suis* biovar 3 strains in this study displayed the same Suis-ladder PCR profiles as that in the corresponding reference strains. Test isolates were further examined by MLVA (Table 2). Two *B. suis* 3 isolates exhibited similar genetic features, namely, MLVA-8 [4 (2-3-4-11-3-1-5-2)] and MLVA-11[31(2-3-4-11-3-1-5−2-4-38-9)], respectively. A comparison of the Ulanqab *B. suis* isolates with the five *B. suis* standard reference strains and vaccine S2 by MLVA-8 was performed. The Ulanqab *B. suis* 3 isolates and *B. suis* biovar 3 reference strains had identical MLVA-8 [4 (2-3-4-11-3-1-5-2)] genotypes, and four loci that differed from those of *B. suis* vaccine S2. The MLVA-11 data were used to trace the geographical source of the test strains comprising human and animal isolates from China. *B. suis* isolates, which exhibited identical MLVA-11 genotypes with strains from four different provinces, namely, Guangxi (*n* = 8), Guangdong (*n* = 2), Hainan (*n* = 1), Beijing (*n* = 1), respectively, of which of two *B. suis* isolated (bru073, bru074) from Guangxi Province were isolated from human and swine, respectively. MLVA-16 showed that these four strains were (ws151, ws152 and bru073, and bru074) different but had closely related genotypes (Figure 1). MLVA analysis suggested that the isolates from Ulanqab and Guangxi Provinces were derived from a common origin and were thus closely related. Comparison of the two *B. suis* isolates of this study with strains from the MLVA bank identified unique genotypes.

**Table 1:** Results of classical biotyping of reference and test strain.

**Strain**	**Growth feature**		**Dye inhibition test**		**Serum agglutination test**				**Phage lysis typing (RTD)**			**Result**
	**CO_2_ requirement**	**H_2_S production**	**Thionin**	**basic fuchsin**	**Positive agglutination**	**A**	**M**	**R**	**Tb**	**BK_2_**	**Wb**
BM 16M	-	–	+	+	+	–	+	–	–	+	–	BM bv. 1
BA 544	±	+	–	+	+	+	–	–	+	+	+	BA bv. 1
BS 1330	–	++	+	–	+	+	–	–	–	+	+	BS bv. 1
WS151	–	–	+	+	+	+	–	–	–	+	+	BS bv. 3
WS152	–	–	+	+	+	+	–	–	–	+	+	BS bv. 3

*BM, Brucella melitensis; BA, Brucella abortus; BS, Brucella suis*.

**Table 2:** Sixteen variable number of tandem repeat loci of two Ulanqab *B. suis* genotypes (WS151, WS152).

**Panel**	**Locus**	***B. suis*** **biovar 3**		***B. suis* biovar *3* reference strains**
		**WS151**	**WS152**
Panel 1	Bruce 06	2	2	2
	Bruce 08	3	3	3
	Bruce 11	4	4	4
	Bruce 12	11	11	11
	Bruce 42	3	3	3
	Bruce 43	1	1	1
	Bruce 45	5	5	5
	Bruce 55	2	2	2
Panel 2	Bruce 18	4	4	4
	Bruce 19	38	38	38
	Bruce 21	9	9	9
	Bruce 04	9	8	7
	Bruce 07	5	5	5
	Bruce 09	7	7	10
	Bruce 16	8	8	4
	Bruce 30	6	3	5

*Detailed information on the B. suis biovar 3 reference strains. Key: REF 686, host: swine, location: United States*.

## Discussion

The present study isolated and characterized *B. suis* biovar 3 isolates from sheep spleens from Inner Mongolia. The detection of Brucella species is essential to the the establishment of pathogen prevalence and disease risk. Inner Mongolia has the top three incidence rate of Brucellosis among Chinese provinces. *B. melitensis* biovar 3 accounts for more than 85% of the total number of examined isolates in the area. Although *B. suis* biovar 3 has been previously reported as the causative pathogen in human brucellosis in Hainan Province (17), no cases of *B. suis* biovar 3 infection in animals or humans were observed in Inner Mongolia in past three decades. Brucella species are phylogenetically closely related (18), and despite differences in host preference, some species are capable of cross-species infections. Consequently, the findings of this study may be beneficial in better understanding *B. suis* transmission patterns between humans and different domestic animals.

In the present study, we used biotyping and MLVA methods to analyze Brucella isolates from sheep in Ulanqab of Inner Mongolia. Although classical biotyping is the gold standard for species and biovar attribution of isolates, it does not accurately identify the species and biovar of atypical *Brucella* spp. (19). MLVA was able to discriminate biovars of most Brucella species and elucidate the relationship among Brucella isolates. However, due to the high genetic diversity of Brucella spp., no single specific method can discriminate all biovars of *Brucella* spp. (16). Therefore, we suggest that classical biotyping and MLVA characterization be utilized as complementary tools for Brucella strain identification and epidemiological analysis.

The two *B. suis* isolates and *B. suis* reference strain biovar 3 shared panel 1 genotype 4 and were distinguished from other *B. suis* reference strains (biovars 1, 2, 4, and 5), differing in one to four loci. Sheep in Inner Mongolia receive the S2 vaccine for 3 consecutive years as part of the national brucellosis control program. Jiang et al. reported that vaccine S2 is genetically highly stable when the strain is passaged 40 times *in vitro* (20). Furthermore*, two* isolates displayed Suis-ladder PCR profiles that were similar to that of the *B. suis* biovar 3 reference strain. This data further confirms that the *B. suis* isolate in this study is distinct from that used in the S2 vaccine.

MLVA-11 has been utilized in tracing the geographical origin of brucellosis infections. *B. suis* biovar 3 isolates in this study have identical MLVA-11 genotypes of strains from four different provinces of China, including Guangxi, Guangdong, Hainan, and Beijing provinces, suggesting that these were possibly derived from a common origin. A previous study confirmed that *B. suis* biovar 3 has only been recently isolated in the Guangdong and Hainan provinces (17). However, brucellosis epidemics involving *B. suis* biovar 3 exclusively occurred in Hainan Province. Furthermore, we observed that *B. suis* biovar 3 strains from the north (Inner Mongolia and Beijing) and south (Guangdong and Guangxi) of China were of MLVA-11 genotype 31, despite originating from different hosts, including humans, swine, and sheep. These data reveal that *B. suis* biovar 3 strains are continuously circulating among different hosts in brucellosis epidemic regions in China.

Previous studies have confirmed the correlation between MLVA-16 genotyping results with epidemiological data, particularly those displaying identical or very closely related genotypes (8, 11, 16, 21, 22). The epidemiological relationship among isolates from different geographical origins in China was evaluated using the MLVA-16 scheme. Two *B. suis* biovar 3 strains from this study (*n* = 2) shared a similar MLVA-16 genotype with strains obtained from Guangxi Province (*n* = 2) and mainly differed in three highly variable loci (bruce04, bruce16, and bruce30) in Panel 2B. They could also be distinguished from most of the other strains from China, thereby suggesting a potential epidemiological correlation among the isolates. The three-locus differencein the highly variable microsatellites from panel 2B reveals that these strains are closely related and presumably originated from a recent common ancestor.

A previous study has reported that the phenomenon of host shifting is uncommon between *B. suis* (swine) and accessory hosts (e.g., sheep, cattle, and spotted deer) (20), although only focusing on brucellosis serology and epidemiology. The present study described the first isolation of *B. suis biovar 3* from sheep spleens in Inner Mongolia, thereby confirming that *B. suis* biovar 3 is capable of shifting hosts between swine and sheep. The findings of the present study suggest that *B. suis* biovar 3 continues to circulate in brucellosis endemic regions with swine and sheep as reservoir hosts and subsequently transmitted to other animals and humans.

## Conclusions

This is the first study that has characterized *B. suis* biovar 3 isolated from sheep spleens in Inner Mongolia that had the MLVA-16 genotype that was similar to strains isolated from different hosts (human and swine) in Guangxi Province. Information on the prevalence and circulation of *Brucella* strains in different livestock species facilitates in understanding its transmission patterns and risk factors for infection. The present study highlights that *B. suis* is capable of host shifts between swine and sheep, eventually infecting humans. MLVA has been utilized to elucidate the biological significance of *B. suis* host shifts between swine and sheep. We suggest that *B. suis* be considered as a significant zoonotic risk to humans and animals in this region and additional strains from wildlife be isolated and characterized.

## Author contributions

ZL performed strain isolation, MLVA typing and cluster analysis, and drafted the manuscript; MW and LW conducted epidemiological investigations and data analysis; HZ and DP prepared the DNA samples; HJ participated in the design of the study and critically reviewed the manuscript; BC and RL participated in the design of the study and also managed the project. All authors have read and approved the final manuscript.

### Conflict of interest statement

The authors declare that the research was conducted in the absence of any commercial or financial relationships that could be construed as a potential conflict of interest.
