# Supplementary material for: Molecular Investigation of the Transmission Pattern of Brucella suis 3 From Inner Mongolia, China
Source: Front Vet Sci. 2018 Oct 29;5:271. doi: 10.3389/fvets.2018.00271 (PMC6215816; doi:10.3389/fvets.2018.00271)
Supplement: Supplementary file 2 [file Table_2.docx]

Table S2. Primer information of MLVA-16 was used in this study

| Locus | Repeat  Unit | 16M  Product  size | 16M Repeat  Unit  number | Sequence（5'-3' ） |
| --- | --- | --- | --- | --- |
| Bruce06 | 134bp | 408bp | 3u | (F) ATGGGATGTGGTAGGGTAATCG |
|  |  |  |  | (R)GCGTGACAATCGACTTTTTGTC |
| Bruce08 | 18bp | 348bp | 4u | (F)ATTATTCGCAGGCTCGTGATTC |
|  |  |  |  | (R)ACAGAAGGTTTTCCAGCTCGTC |
| Bruce11 | 63bp | 257bp | 2u | (F)CTGTTGATCTGACCTTGCAACC |
|  |  |  |  | (R)CCAGACAACAACCTACGTCCTG |
| Bruce12 | 15bp | 392bp | 13u | (F)CGGTAAATCAATTGTCCCATGA |
|  |  |  |  | (R)GCCCAAGTTCAACAGGAGTTTC |
| Bruce42 | 125bp | 539bp | 4u | (F)CATCGCCTCAACTATACCGTCA |
|  |  |  |  | (R)ACCGCAAAATTTACGCATCG |
| Bruce43 | 12bp | 182bp | 2u | (F)TCTCAAGCCCGATATGGAGAAT |
|  |  |  |  | (R)TATTTTCCGCCTGCCCATAAAC |
| Bruce45 | 18bp | 151bp | 3u | (F)ATCCTTGCCTCTCCCTACCAG |
|  |  |  |  | (R)CGGGTAAATATCAATGGCTTGG |
| Bruce55 | 40bp | 273bp | 3u | (F)TCAGGCTGTTTCGTCATGTCTT |
|  |  |  |  | (R)AATCTGGCGTTCGAGTTGTTCT |
| Bruce04 | 8bp | 152bp | 2u | (F)CTGACGAAGGGAAGGCAATAAG |
|  |  |  |  | (R)CGATCTGGAGATTATCGGGAAG |
| Bruce07 | 8bp | 158bp | 5u | (F)GCTGACGGGGAAGAACATCTAT |
|  |  |  |  | (R)ACCCTTTTTCAGTCAAGGCAAA |
| Bruce09 | 8bp | 156bp | 7u | (F)GCGGATTCGTTCTTCAGTTATC |
|  |  |  |  | (R)GGGAGTATGTTTTGGTTGTACATAG |
| Bruce16 | 8bp | 152bp | 3u | (F)ACGGGAGTTTTTGTTGCTCAAT |
|  |  |  |  | (R)GGCCATGTTTCCGTTGATTTAT |
| Bruce18 | 8bp | 146bp | 5u | (F)TATGTTAGGGCAATAGGGCAGT |
|  |  |  |  | (R)GATGGTTGAGAGCATTGTGAAG |
| Bruce19 | 6bp | 163bp | 18u | (F)GACGACCCGGACCATGTCT |
|  |  |  |  | (R)ACTTCACCGTAACGTCGTGGAT |
| Bruce21 | 8bp | 148bp | 6u | (F)CTCATGCGCAACCAAAACA |
|  |  |  |  | (R)GATCTCGTGGTCGATAATCTCATT |
| Bruce30 | 8bp | 151bp | 6u | (F)TGACCGCAAAACCATATCCTTC |
|  |  |  |  | (R)TATGTGCAGAGCTTCATGTTCG |
